# Responses to dichotic tone-in-noise stimuli in the inferior colliculus

**DOI:** 10.3389/fnins.2022.997656

**Published:** 2022-12-01

**Authors:** Langchen Fan, Kenneth S. Henry, Laurel H. Carney

**Affiliations:** ^1^Department of Biomedical Engineering, University of Rochester, Rochester, NY, United States; ^2^Department of Neuroscience, University of Rochester, Rochester, NY, United States; ^3^Department of Otolaryngology, University of Rochester, Rochester, NY, United States

**Keywords:** binaural masking level difference, binaural cues, binaural detection, interaural correlation, midbrain

## Abstract

Human listeners are more sensitive to tones embedded in diotic noise when the tones are out-of-phase at the two ears (N_0_S_π_) than when they are in-phase (N_0_S_0_). The difference between the tone-detection thresholds for these two conditions is referred to as the binaural masking level difference (BMLD) and reflects a benefit of binaural processing. Detection in the N_0_S_π_ condition has been explained in modeling studies by changes in interaural correlation (IAC), but this model has only been directly tested physiologically for low frequencies. Here, the IAC-based hypothesis for binaural detection was examined across a wide range of frequencies and masker levels using recordings in the awake rabbit inferior colliculus (IC). IAC-based cues were strongly correlated with neural responses to N_0_S_π_ stimuli. Additionally, average rate-based thresholds were calculated for both N_0_S_0_ and N_0_S_π_ conditions. The rate-based neural BMLD at 500 Hz matched rabbit behavioral data, but the trend of neural BMLDs across frequency differed from that of humans.

## Introduction

Human listeners benefit from binaural hearing in detection tasks. For example, in the tone-in-noise (TIN) detection task, the threshold for detection of out-of-phase tone in identical noise at the two ears (N_0_S_π_) is lower (i.e., better) than that for detection of an in-phase tone (N_0_S_0_) (e.g., Hirsh, 1948; Hawley et al., 2004). The difference in detection thresholds between the N_0_S_0_ and N_0_S_π_ conditions is referred to as the binaural masking level difference (BMLD).

In N_0_S_π_ stimuli, the difference between the tone-plus-noise waveforms at the two ears results in differences in interaural time or phase and level differences (ITDs, IPDs, or ILDs), as well as changes in the interaural correlation (IAC) (e.g., Domnitz and Colburn, 1976; Bernstein and Trahiotis, 1996). The statistics of the interaural phase and level cues, and their distributions for different signal-to-noise ratios (SNRs) for stimuli used in binaural detection experiments are described in Zurek (1991). Experiments designed to distinguish the relative importance of dynamic ITD vs. IAC cues have suggested that ITD is most important for 500-Hz binaural detection (van der Heijden and Joris, 2010). Furthermore, a psychophysical study that manipulated ITD and IAC cues over a wide range of frequencies showed that predictions for tone detection differ for ITD and IAC cues (Culling, 2011), and as expected, the role of the ITD cue is diminished at higher target frequencies. The challenge of discriminating between models based on these cues, which co-vary in stimuli used for binaural detection, was described by Domnitz and Colburn (1976), who stressed the importance of testing these models over a range of frequencies or other stimulus parameters in order to distinguish the models. Several subsequent models for binaural detection have focused on detection of a decrease in IAC upon addition of a tone in the N_0_S_π_ condition and have tested this class of model across a wide range of stimulus conditions (e.g., Colburn, 1977; Bernstein and Trahiotis, 1997, 2017).

Human listeners can have substantial BMLDs (>3 dB) up to at least 8 kHz (van de Par and Kohlrausch, 1999; Goupell, 2012), yet physiological studies have mainly focused on low frequencies, for which the BMLD is typically larger (up to 20 dB, depending on bandwidth) (e.g., van de Par and Kohlrausch, 1999). Early physiological studies of detection of tones in N_0_S_π_ stimuli focused on sensitivity of low-frequency neurons in the auditory midbrain (inferior colliculus, IC) to ITDs (e.g., Caird et al., 1991; McAlpine et al., 1996; Jiang et al., 1997a,b). Later physiological studies analyzed low-frequency IC responses in terms of the IAC cue (Palmer et al., 1999; Lane and Delgutte, 2005), and the effect of decorrelation was estimated over a wider frequency range in the owl (Asadollahi et al., 2010). The current study extends this work by applying an analysis of IAC cues to responses in the IC of awake rabbit across a wide range of frequencies. If interaural decorrelation explains neural responses to N_0_S_π_ stimuli, then the difference in average rate between IC responses to diotic noise and binaurally uncorrelated noise should be correlated to the rate difference between responses to the noise-alone condition and the noise-plus-dichotic-tone condition. This correlation was directly tested in this study.

Additionally, human psychophysical studies have shown that BMLDs are robust across a range of noise levels (Buss et al., 2003) and in a roving-level paradigm, in which stimulus level was randomly varied from interval to interval (Henning et al., 2005). Therefore, in the current study neural responses were recorded over a wide range of noise levels to explore trends across sound level.

The IC is a nearly obligatory synapse along the ascending auditory pathway, thus all information available for perception must be encoded at this level. This fact makes the IC an interesting place to examine the relationship between neural and behavioral response properties in tasks such as masked detection. The IC receives afferent inputs from nearly all of the auditory brainstem nuclei (Cant and Oliver, 2018). IC neurons are sensitive to several features of stimuli, including ITDs and ILDs (Reviewed in Yin et al., 2019) and envelope frequency and depth (e.g., Langner and Schreiner, 1988; Krishna and Semple, 2000; Nelson and Carney, 2007; Zheng and Escabi, 2013). Addition of a dichotic tone to a diotic noise masker influences all of these cues. However, individual IC responses are complex in that each neuron responds to different cues with different sizes and directions of rate changes. In the current study, the sensitivities of individual neurons were evaluated using standard physiological characterizations, such as modulation transfer functions and responses to noise with ITDs and ILDs. Responses were then tested for their correlation to the IAC cue. Consistent with previous physiological and psychophysical studies, our results support the importance of the IAC in shaping IC responses to stimuli used to estimate BMLDs, and extend these results by illustrating that this correlation extends across a wide range of noise levels and frequencies.

The current study also computed rate-based IC neural thresholds for comparison with published detection thresholds for human listeners (van de Par and Kohlrausch, 1999; Buss et al., 2003; Goupell, 2012) and rabbits (Zheng et al., 2002).

## Materials and methods

All neurophysiological procedures were approved by the University of Rochester Committee on Animal Resources. Recordings were from four awake, female Dutch-belted rabbits with normal hearing. Distortion product otoacoustic emissions (Whitehead et al., 1992) were used to monitor hearing over the timecourse of the study. Two of the rabbits were studied from 17 to 55 months of age, and two rabbits from age 13 to 23 months.

### Procedures

Surgical and recording procedures are described in detail in Fan et al. (2021). Briefly, rabbits were anesthetized with an intramuscular injection of ketamine (66 mg/kg) and xylazine (2 mg/kg) for both headbar placement and microdrive (five-drive, Neuralynx, Inc., Bozeman, MT, USA) implantation surgeries. The headbar was custom-designed, 3D-printed hard plastic, with a chamber that held the microdrive. The headbar was permanently mounted on the rabbit skull with stainless-steel screws and dental acrylic. After the rabbit recovered from the headbar surgery, a craniotomy was made to allow insertion of guidetubes from the microdrive through the dura. One microdrive held four guidetubes and tetrodes and allowed for independently advancing and retracting each tetrode. Each tetrode consisted of four twisted 18-μm platinum iridium wires, coated in epoxy (California Fine Wire Co., Grover Beach, CA, USA). The microdrive was replaced as needed, with guidetube positions varied across placements, to search for new neurons.

During recording sessions, the rabbit was placed in a double-walled, sound-proof chamber (Acoustic Systems, Austin, TX, USA), with head fixed using the headbar. Sound was delivered using Beyerdynamic DT990 (Beyerdynamic GmbH & Co., Heilbronn, Germany) or Etymotic ER2 earphones (Etymotic Research, Inc., Elk Grove Village, IL, USA) with custom ear molds for each rabbit. Ear molds were positioned deep in the concha and included an Etymotic probe tube for calibration. The stimulus system included an audio interface (16A, MOTU, Cambridge, MA, USA), a digital-to-analog converter (DAC3 HGC, Benchmark Media Systems, Inc., Syracuse, NY, USA), and earphones (Beyerdynamic DT990, Beyerdynamic GmbH and Co., Heilbronn, Germany or ER2, Etymotic Research). Wideband noise bursts were presented to search for auditory responses. Recordings were made with a multi-channel system (RHD, Intan Technologies, LLC., Los Angeles, CA, USA). When the characteristic frequencies (CFs) increased with tetrode depth, the tetrodes were determined to be in the central nucleus of the IC (ICC). Action potentials were identified offline using spike-sorting techniques applied to the tetrode recordings (Schwarz et al., 2012; Fan et al., 2021). After the termination of recording sessions in each animal, post-mortem histology was applied to verify tetrode locations in the IC.

### Stimuli

Speakers were calibrated with ER-7C or ER-10B+ microphones (Etymotic Research) at the beginning of each recording session. The neurons were characterized in several ways before presenting TIN stimuli. Binaural sensitivity was determined by responses to contralateral, ipsilateral, and binaural wideband noise (0.1–19 kHz) at several sound levels. Responses to contralateral pure tones between 0.25 and 20 kHz from 10 to 70 dB SPL were used to identify CF, the frequency at which the neuron responded at the lowest sound level. Noise delay functions (NDFs) described rate responses to noise stimuli as a function of ITD; NDFs were recorded with wideband noise (0.1–19 kHz), 1-sec duration, 30-dB SPL spectrum level, and ITDs from –2,000 to 2,000 μs with a 200-μs stepsize. Responses to ILDs were recorded with the same noise bandwidth and duration as for the NDF. ILDs ranged from –15 to 15 dB with a 5-dB stepsize; the stimulus on the contralateral side had a fixed spectrum level of 30 dB re 20 μPa. Responses to contralateral sinusoidally-amplitude-modulated (SAM) wideband noise (0.1–19 kHz), with 1-sec duration, were collected to identify the shape of the modulation transfer function (MTF). SAM noises were described by:


$$s=[1+\sin(2\pi f_{m}t)]\;n(t)$$

where *n*(*t*) is the wideband noise with a spectrum level of 30 dB SPL, and *f_m_* is the modulation frequency. Modulation frequencies were logarithmically spaced between 2 and 350 Hz, with three steps/octave. Responses to contralateral unmodulated noise were also recorded. For all of the above characterizations, three repetitions of each stimulus condition were presented, in random sequence.

For TIN stimuli, the tone frequency and the center frequency of 1/3-oct gaussian noise maskers were chosen to be approximately equal to CF. Noise maskers were simultaneously gated with tone signals and generated by filtering wideband noise with a 5,000th-order FIR band-pass filter. TIN stimuli had 0.3-sec duration with 10-msec cos^2^ on/off ramps. Overall noise levels ranged from 35 to 75 dB SPL, with a10-dB stepsize. Signal-to-noise ratio (SNR) ranged from –12 to 8 dB, with a 4-dB stepsize; a noise-alone condition was also included. Tone levels and noise levels were presented in random order, and the order was shuffled for each of the 30 repetitions of the stimulus set. Responses were collected for sets of random noise, or reproducible noise (for the temporal analyses in Fan et al., 2021), or both. If more than one dataset was recorded, the dataset with responses to random noise waveforms was used for the analyses presented here. Among all neurons reported here, there were 55 neurons studied with random noise and 81 neurons studied with reproducible noise. No qualitative differences were observed between these two types of datasets, although the use of random noise would be expected to reduce the potential effect of external noise on neural responses.

To test the influence of IAC on IC neurons, responses to diotic (N_0_) and binaurally uncorrelated (N_*u*_) noise were recorded. For both N_0_ and N_*u*_ conditions, the stimuli were 1/3-octave random gaussian noise, with 2-sec duration, at 65 dB SPL. Five repetitions of five N_0_ and ten N_*u*_ noise were presented, in random sequence.

### Noise delay function shape classification

The shape of the NDF, the best ITD (*d*_*BITD*_), and the frequency of ITD tuning (*f*_*ITD*_) were determined by fitting the NDF with a Gabor function (Lane and Delgutte, 2005), a sinusoid modulated by a gaussian function:


$$G_{1}=\left|A\,e^{-\frac{{\left(d_{I\,T\,D}-d_{B\,I\,T\,D}\right)}^{2}}{2\,\sigma^{2}}}\,\cos\left[2\,\pi \,f_{I\,T\,D}\,\left(d_{I\,T\,D}-d_{B\,I\,T\,D}\right)\right]+B\right|,$$

where *d*_*ITD*_ is the interaural delay, A, B, and σ are parameters for the amplitude, DC offset, and standard deviation of the gaussian function, respectively, and |•| refers to half-wave rectification. If a neuron’s CF was more than twice *f*_*ITD*_ (i.e., a high-frequency neuron), indicating that the neuron did not have fine-structure-based ITD sensitivity, then *f*_*ITD*_ was set to zero, and the NDF was refitted with the following gaussian function:


$$G_{2}=\left|A\,e^{-\frac{{\left(d_{I\,T\,D}-d_{B\,I\,T\,D}\right)}^{2}}{2\,\sigma^{2}}}+B\right|.$$

The function was fit to an NDF using a least-square fit, obtained with a trust-region-reflective algorithm (*lsqcurvefit* in MATLAB).

**FIGURE 1:**
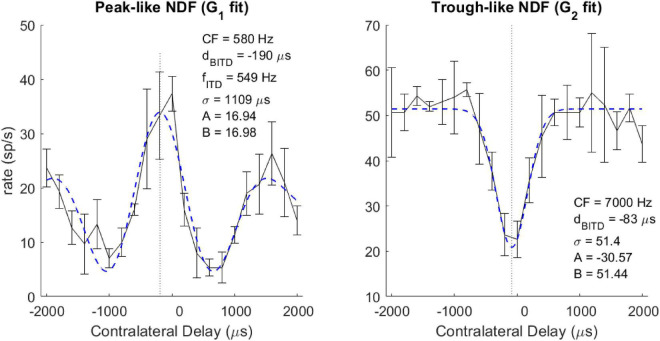
Example neural ITD responses (black solid curve) and fitted Gabor function (blue dashed curve) for peak-like **(left)** and trough-like **(right)** NDFs. Vertical dotted line indicates the best ITD. The neuron’s CF, Best or Worst ITD (*d*_*BITD*_), and for cyclic ITD curves, the ITD tuning frequency (*f*_*ITD*_) are described in the text.

Each NDF was classified as peak-like, trough-like, or ITD-insensitive. In the following cases, the neuron was considered sensitive to ITDs: (1) for NDFs fitted with *G_1_*, if the absolute value of the amplitude (A) was more than 5 spikes/sec; (2) for NDFs fitted with *G_2_*, if the absolute value of the prominence (A/B) was more than 0.25; (3) for NDFs fitted with *G_2_*, for a fit with σ between 60 and 1,000 μs. If the amplitude (A) was positive, the neuron was classified as having a peak-like NDF; otherwise, the neuron was classified as having a trough-like NDF. Other neurons were classified as ITD-insensitive. The classification of each NDF generally agreed with a qualitative assessment (Figure 1).

### Modulation transfer function shape classification

The MTF shape was classified with rules designed to be simple and to agree with qualitative descriptions of the functions. Enhancement or suppression was identified with the Mann-Whitney test as significantly higher or lower rates at two or more neighboring modulation frequencies than the rates in response to unmodulated noise. The presence or absence of enhancement or suppression was used to classify the MTF into the following four types: all-pass (AP, no enhancement or suppression), band-enhanced (BE, only enhancement), band-suppressed (BS, only suppression), and hybrid (both enhancement and suppression, over different ranges of modulation frequency).

### Rate analysis

Average rates, excluding 20-ms onset responses, were calculated for responses to all stimuli. For TIN stimuli, at each noise and tone level (i.e., SNR), a rate-based receiver-operating-characteristic (ROC, Egan, 1975) was calculated using average rate responses for all 30 noise-alone and tone-plus-noise presentations. The percent-correct performance was estimated from the area under the ROC curve. Note that rates in response to tone-plus-noise stimuli could be either higher or lower than rates in response to noise-alone stimuli, so the minimum percent correct was limited to 50%, regardless of the direction of change in rate. The neural threshold was estimated using linear interpolation to find the lowest SNR with 70.7% correct, which corresponds to a threshold estimated with a two-down, one-up tracking procedure (Levitt, 1971).

## Results

**FIGURE 2:**
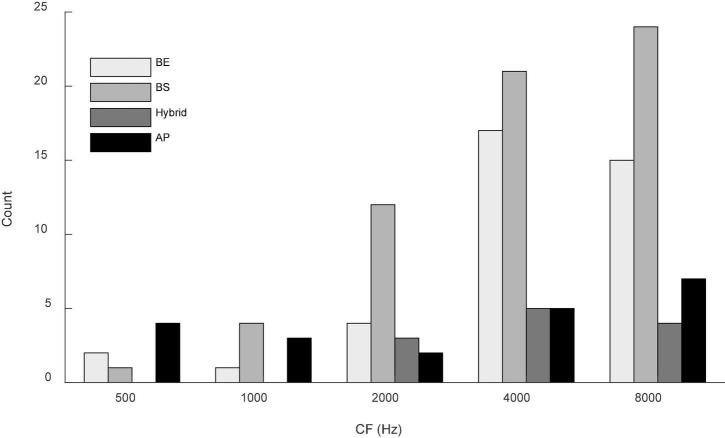
Distribution of MTFs across CF (in one-octave bins) for the units presented in this study. Gray shades from light to dark indicate units with band-enhanced (BE), band-suppressed (BS), hybrid and all-pass (AP) MTF shapes. Two neurons with CF of 12.1k were included in the last bin for simplicity. Most MTF types were represented across the range of CFs, although hybrid MTFs were not observed at the lower CFs.

Responses to both N_0_S_0_ and N_0_S_π_ stimuli were recorded from 136 isolated single units; responses to N_0_S_0_ of 111 of these units were presented in Fan et al. (2021). Responses to Nu were recorded for 68 units. The distribution of CFs is shown in Figure 2. All units were tested using a tone frequency within 1/3-octave of the neuron’s CF. Based on the MTF categorization criteria described above, there were 40 BE units (29.4%), 62 BS units (45.6%), 12 hybrid units (8.8%) and 22 AP units (16.2%). Distribution of MTF types across CFs is shown in Figure 2.

### Examples of single-neuron responses

**FIGURE 3:**
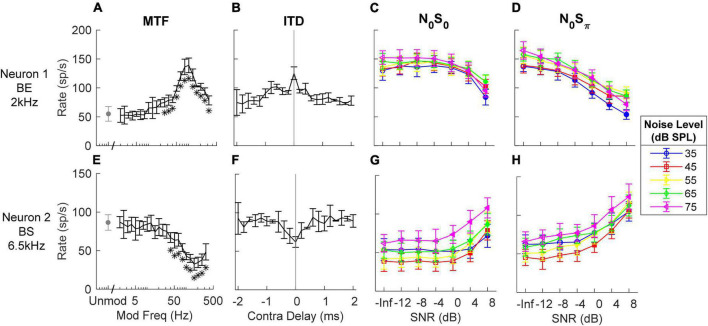
Responses of two example neurons (**top** and **bottom** row respectively). **(A,E)** MTF, response rates to amplitude-modulated noise; stars indicate modulation frequencies that had rates significantly different from the unmodulated condition. **(B,F)** ITD sensitivity, response rates vs. time delay in contralateral side (negative indicates ipsilateral side has delay). **(C,D,G,H)** responses to N_0_S_0_ and N_0_S_π_ stimuli at different noise levels (different symbols) vs. SNR (from left to right); filled symbols indicate supra-thresholds. Errorbars indicate standard deviation. MTF shape and tone frequency for TIN stimuli (close to CF) are shown on the left. The example BE neuron had decreasing rate upon addition of a tone for both N_0_S_0_ and N_0_S_π_, while the example BS neuron had increasing rate for both conditions.

Responses of several example units illustrate the complexity of response properties of the IC responses that were analyzed to test the IAC hypothesis. IC neurons have rates that vary with both ITD and ILD, and the interaction of these cues in the N_0_S_0_ and N_0_S_π_ stimuli are complex (Zurek, 1991). Additionally, IC neurons are sensitive to periodicity in the stimulus as conveyed in their neural inputs. Adding a tone to narrowband gaussian noise flattens the stimulus envelope (Richards, 1992) and also reduces the amplitudes of neural fluctuations in peripheral responses (Carney, 2018). Therefore, the MTFs of IC neurons are interesting to consider, as well as sensitivity to the classical interaural cues. Neurons with BE MTFs (Figure 3A) are excited by fluctuations and therefore expected to have decreasing rate with increasing SNR for TIN stimuli. On the contrary, neurons with BS MTFs (Figure 3E) are suppressed by fluctuations and therefore expected to have increasing rate with increasing SNR. As expected, Neuron 1, with a BE MTF, had decreasing rate versus SNR at all noise levels (Figures 3C,D), and Neuron 2, with a BS MTF, had increasing rate versus SNR at all noise levels (Figures 3G,H). Both of these examples responded as predicted by their MTF types. Note that for both neurons in Figure 3, the average rate changed at lower SNRs for the N_0_S_π_ condition than for the N_0_S_0_ condition, for all noise levels tested, indicating lower neural thresholds, consistent with psychophysical results (e.g., van de Par and Kohlrausch, 1999).

Neural responses to N_0_S_0_ stimuli have previously been described as having increasing rate as a function of tone level (Jiang et al., 1997a; Ramachandran et al., 2000), possibly based on the assumption that neurons respond more strongly to increasing stimulus energy (i.e., upon addition of a tone). Note that Neuron 1 in Figure 3 is an example of a neuron that had decreasing rate as tone level increased at each masker level, whereas it had increasing rate versus masker level for the noise-alone stimuli (SNR = –inf); these responses cannot be explained based on stimulus energy. The shape of NDF has been used to explain changes in neural responses for the N_0_S_π_ condition (Jiang et al., 1997a,b): a diotic noise masker has zero ITD; adding a dichotic tone introduces non-zero ITDs. Neurons with peak-like NDFs respond most strongly to near-zero ITDs, and thus would be expected to have decreasing rate with increasing SNR based on the ITD hypothesis. In contrast, neurons with trough-like NDFs would be expected to have increasing rate with increasing SNR. Responses to N_0_S_π_ stimuli of Neurons 1 and 2 can also be explained by their NDF shapes: Neuron 1 had a peak-like NDF shape (Figure 3B) and decreasing rate versus SNR for the N_0_S_π_ condition; Neuron 2 had a trough-like NDF (Figure 3F) and increasing rate versus SNR.

Single-unit responses to N_0_S_0_ and N_0_S_π_ stimuli were analyzed based on MTF properties and responses to ITDs and ILDs. In general, the directions and sizes of rate differences to N_0_S_0_ stimuli can be predicted based on MTF properties (Fan et al., 2021), but in response to N_0_S_π_ stimuli, predictions of changes in rate based on MTF properties were only significant at the highest noise level tested (Fan, 2020). Rate differences were also weakly but significantly correlated to rate differences in the NDF, but the correlations decreased as stimulus level increased (Fan, 2020).

**FIGURE 4:**
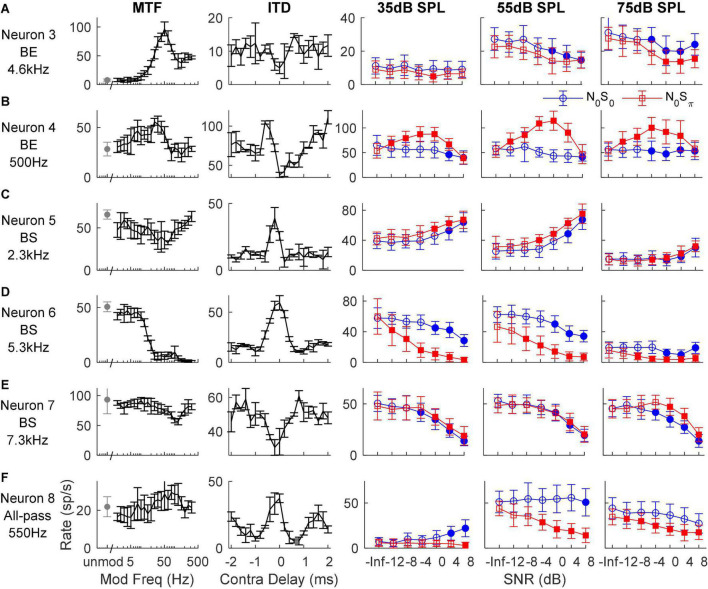
Responses of six example neurons **(A–F)**. The left two columns show the neuron’s MTF and ITD sensitivity, respectively. The right three columns show the neuron’s response to N_0_S_0_ (blue circles) and N_0_S_π_ (red squares) TIN stimuli at noise levels of 35, 55, and 75 dB SPL, respectively; filled symbols indicate supra-threshold responses. MTF shape and tone frequency of TIN stimuli (close to CF) are shown on the left.

In general, IC responses to dichotic TIN stimuli are not easily explained by characterizations based on MTFs, ITDs, or ILDs (see below), likely because of the interaction of these cues in N_0_S_0_ and N_0_S_π_ stimuli and because of the different types of sensitivity of IC neurons to these cues (Figure 4). For example, Neurons 3 and 4 both had BE MTFs and decreasing rate versus SNR for the N_0_S_0_ condition at most noise levels, as expected. However, for the N_0_S_π_ condition, Neuron 3 had decreasing rate versus SNR that could be explained by its MTF shape, but not its trough-like NDF. In contrast, Neuron 4 had an increasing rate versus SNR that could be explained by its NDF shape, but not by its MTF shape. Neurons 5, 6, and 7 all had BS MTFs, and thus were expected to have increasing rates versus SNR, but the responses of these neurons differ. Neuron 5 had increasing rate versus SNR for both N_0_S_0_ and N_0_S_π_ conditions, which could be explained by its BS MTF, but not by its peak-like NDF. The MTF of Neuron 6 did not explain responses to either N_0_S_0_ or N_0_S_π_ stimuli, but responses to N_0_S_π_ stimuli (decreasing rate) could be explained by its peak-like NDF. Neuron 7 also had decreasing rate versus SNR, which could not be explained by either MTF or NDF shape. Neuron 8 had an all-pass MTF, and responses to N_0_S_π_ stimuli that could be explained by its peak-like NDF.

### Rate differences in response to N_0_S_π_ stimuli and binaural cues

**FIGURE 5:**
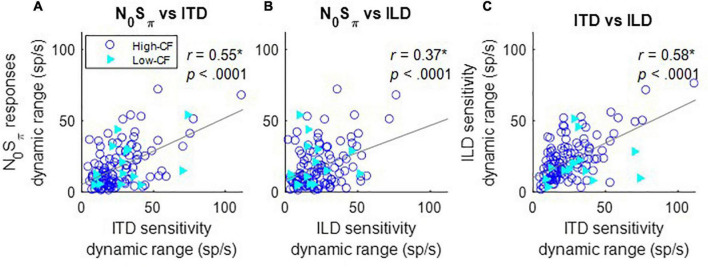
Correlation between dynamic ranges of responses to N_0_S_π_ and ITD **(A)**, N_0_S_π_ and ILD **(B)**, and ILD and ITD **(C)** at 65 dB SPL (as indicated in titles). Correlation coefficients and *p*-values are shown at the top right of each panel; a star indicates that the correlation coefficient was significant after Bonferroni correction (*p* < 0.017). Neurons with CF below 1.5 kHz (low–CF) are shown with filled triangles, whereas neurons with CF above 1.5 kHz (high–CF) are shown with open circles. Solid gray lines indicate linear regressions.

The rate differences in response to ITDs or ILDs were quantified by the difference between the maximum and minimum response rates over the range of stimuli tested. The maximum change in rate in response to N_0_S_π_ stimuli, for both directions of rate change as a function of SNR, was significantly correlated to the maximum rate differences in both ITD and ILD responses (Figures 5A,B), explaining a small but significant proportion of the variance (i.e., *r*^2^). There was not an obvious difference between results shown in Figure 5 for lower-CF neurons (< 1.5 kHz, filled triangles) vs. higher-CF neurons (open circles). The significant correlation between the maximum rate differences for N_0_S_π_ responses and rate differences for both ITD and ILD responses could be because (1) adding a dichotic tone not only introduces ITDs, but also ILDs; and/or (2) the dynamic ranges of ITD and ILD responses were significantly correlated (Figure 5C). Changes in neural responses to N_0_S_π_ are likely due to a combination of ITD and ILD sensitivities and to the co-variation of these cues. The standard deviations of interaural phase and interaural level cues as a function of SNR have been previously described [see Figures 9 and 10 in Zurek (1991)].

### Inferior colliculus responses to interaural correlation

**FIGURE 6:**
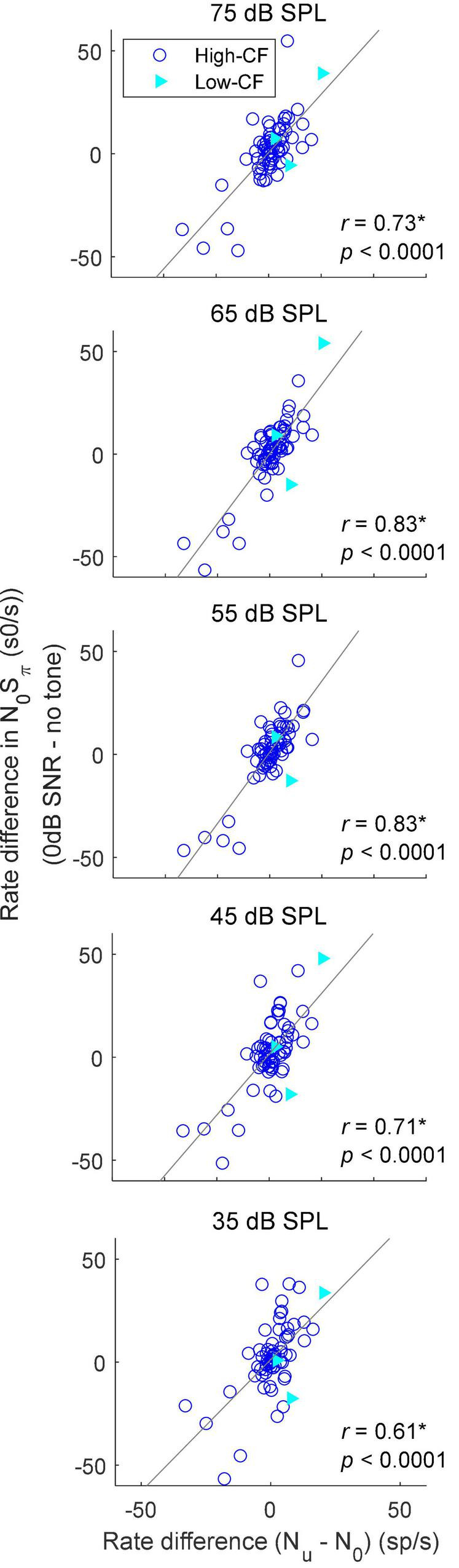
Correlation between the rate difference elicited by addition of a dichotic tone (N_0_S_π_) at 0 dB SNR and the rate difference between responses to N_0_ and N_u_ conditions. Correlation coefficients and *p*-values are shown; a star indicates that the correlation coefficient was significant after Bonferroni correction (*p* < 0.0014). Neurons with CF below 1.5 kHz (low–CF) are shown in filled triangles, whereas neurons with CF above 1.5 kHz (high–CF) are shown in open circles. Solid lines show linear regressions.

Adding a dichotic tone to diotic noise introduces both ITD and ILD cues, as well as interaural decorrelation, but the changes in these cues differ for different tokens of noise waveform as well as for different SNRs. For example, the ITD of a N_0_S_π_ stimulus is dominated by the ITD of the added tone with increasing tone level, but the effective ITD of a N_0_S_π_ stimulus with a low-SNR tone (e.g., at threshold) is hard to estimate, and varies with the noise token due to the phase interaction between the noise and tone. Additionally, unlike a pure tone, the instantaneous ITD of N_0_S_π_ stimuli varies throughout the duration of the stimulus waveform. Therefore, prediction of the rate-change direction upon addition of a tone at threshold based on sensitivity to static ITDs and ILDs is not simple. On the other hand, the effect of interaural decorrelation can be studied with a more straightforward method. To examine the effect of decorrelation, average rates were recorded in response to 1/3-octave diotic (N_0_) and binaurally uncorrelated (N_*u*_) gaussian noise for 68 neurons. The N_*u*_ noises presented at the two ears were simply independent narrowband noise tokens. The correlation between the difference in average rate in response to the N_0_S_π_ condition (the difference between average rates in response to noise-alone and to N_0_S_π_ at 0-dB SNR) and the difference in average rates in response to the N_0_ and N_*u*_ conditions was significant at all noise levels (Figure 6), supporting the hypothesis that IC rates are influenced by IAC. The correlation was strongest for TIN stimuli with a masker level of 65 dB SPL, the level at which the N_0_ and N_*u*_ noise were presented. At 65 dB SPL, additional analyses of the rate differences in responses to N_0_S_π_ stimuli at SNRs of –8 to 8 dB relative to the noise-alone condition were all significantly correlated to the rate difference between the responses to N_*u*_ and N_0_ noise, with correlation coefficients ranging from 0.71 to 0.84, and *p* values all less than 0.0001 (significant after Bonferroni correction, not shown). The significant correlation coefficients at *all* SNRs and noise levels indicated that, in general, the direction and size of the changes in rate in response to N_0_S_π_ stimuli were explained by the change in the stimulus from N_0_ toward N_*u*_. Note that there were only a few low-CF (<1.5 kHz, filled triangles) in this dataset, so it is clear that the correlations illustrated in Figure 6 applied to the much larger group of high-CF neurons (open circles).

### Rate-based neural thresholds

**FIGURE 7:**
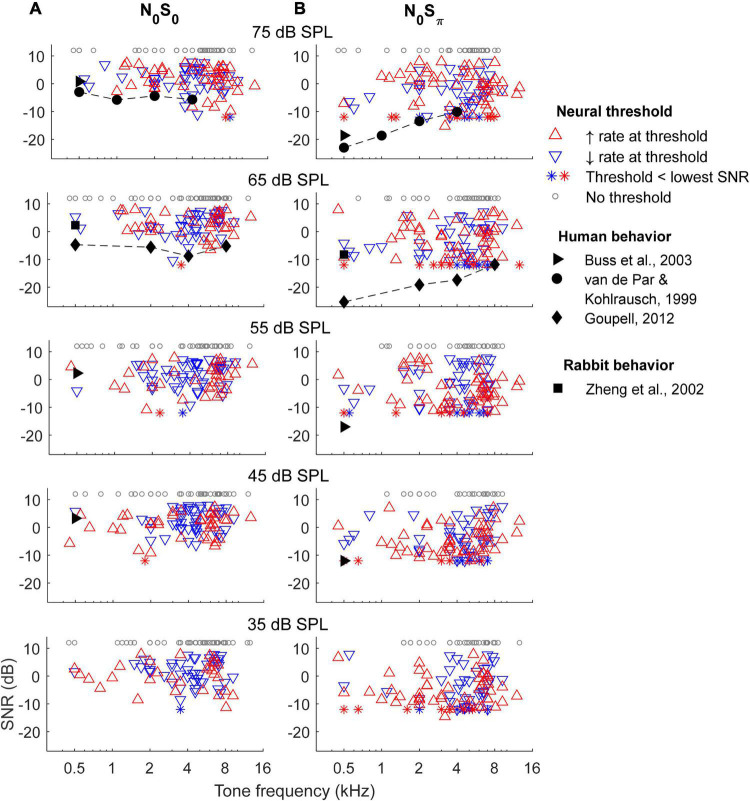
Rate-based threshold for N_0_S_0_ **(A)** and N_0_S_π_ **(B)** conditions. Thresholds of most sensitive neurons across frequencies matched human behavioral data for the N_0_S_0_ condition, but had a trend different from human for the N_0_S_π_ condition. Neural thresholds at 500 Hz matched rabbit behavioral data for both conditions.

Rate-based thresholds of all units for the N_0_S_0_ and N_0_S_π_ conditions at five noise levels were computed and compared with behavioral data from previous studies (Figure 7). There was no clear trend in the numbers of units with increasing or decreasing rate-change direction across frequency, for either the N_0_S_0_ or N_0_S_π_ condition, except a weak trend of more units with increasing rate at the lowest noise level tested (bottom row). The lowest rate thresholds across frequency were lower for the N_0_S_π_ condition than for the N_0_S_0_ condition, as expected.

The lowest rate thresholds at 500 Hz matched the mean rabbit behavioral detection threshold at the same frequency (Zheng et al., 2002). Compared with human thresholds, the lowest rate thresholds for the N_0_S_0_ condition were close to human thresholds across frequencies, but the lowest rate thresholds for the N_0_S_π_ condition only matched human thresholds at high frequencies (note that the lower limit of SNRs tested limited this comparison, see below). Human thresholds from Goupell (2012) are slightly lower than van de Par and Kohlrausch (1999) at some frequencies, possibly due to differences in paradigm and stimulus bandwidths. Note that stimuli used in previous studies have slightly different parameters from this study: stimuli in Zheng et al. (2002) had 200-Hz bandwidth (vs. 116 Hz in this study) and an overall level of 63 dB SPL; stimuli in van de Par and Kohlrausch (1999) had bandwidths of 100, 250, 500 Hz and 1 kHz (vs. 116 Hz, 232, 463, and 926 Hz in this study) for center frequencies of 500 Hz, 1, 2, and 4 kHz, and with overall level of 70 dB SPL; stimuli in Buss et al. (2003) had 50-Hz bandwidth and overall noise levels of 42, 57, and 72 dB SPL; stimuli in Goupell (2012) had bandwidths of 78, 240, 456, and 888 Hz (vs. 116, 463, 926, and 1,852 Hz in this study) for center frequencies of 500 Hz, 2, 4, and 8 kHz. However, despite the discrepancies among stimuli, in general, the lowest rate-based thresholds could explain human thresholds for the N_0_S_0_ condition across all frequencies tested and for the N_0_S_π_ condition at high frequencies. Note that the thresholds of most sensitive neurons across frequencies did not vary qualitatively across noise levels, consistent with human thresholds tested at multiple noise levels (Buss et al., 2003) and with a roving-level paradigm (Henning et al., 2005).

### Rate-based neural binaural masking level differences

**FIGURE 8:**
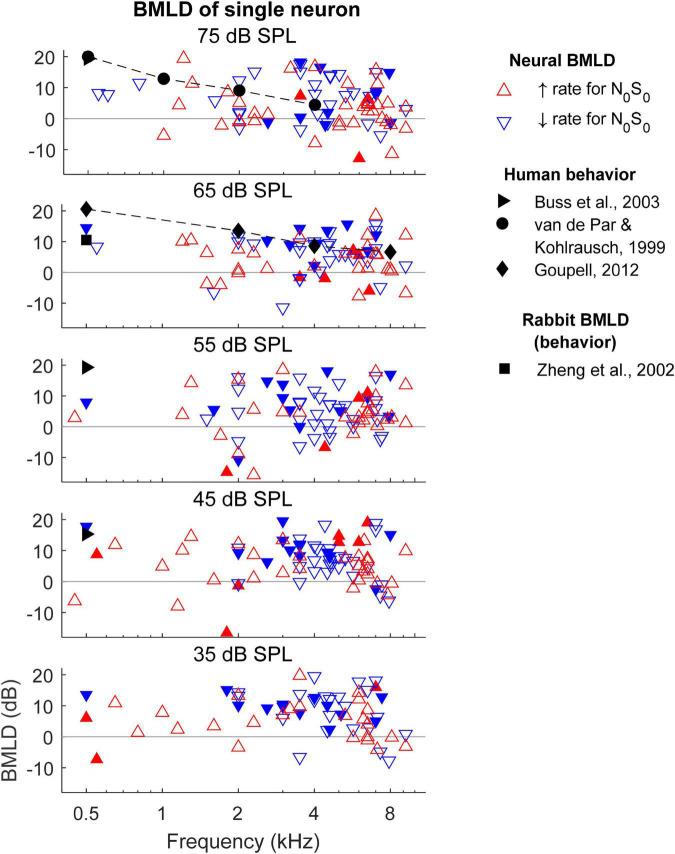
Binaural masking level differences (BMLDs) calculated based on single-neuron thresholds for both N_0_S_0_ and N_0_S_π_ conditions. Open triangles indicate that the direction of change in rate vs. SNR at threshold for the N_0_S_π_ condition was the *same* as for the N_0_S_0_ condition, whereas filled triangles indicate *opposite* direction of change in rate at threshold for the N_0_S_0_ and N_0_S_π_ conditions. Only neurons that had measurable thresholds in both N_0_S_0_ and N_0_S_π_ conditions are shown here.

Neural BMLDs were evaluated in two ways: using the BMLDs of individual neurons, and using the BMLDs calculated from the N_0_S_0_ and N_0_S_π_ thresholds of the neural population. For BMLDs of single neurons (Figure 8), only neurons with measurable thresholds for both N_0_S_0_ and N_0_S_π_ conditions are plotted, together with human BMLDs (van de Par and Kohlrausch, 1999; Buss et al., 2003; Goupell, 2012). BMLDs were typically positive, indicating greater TIN sensitivity for N_0_S_π_ compared to N_0_S_0_. There was no clear association observed between small or negative.

BMLDs and rate-change direction for either N_0_S_0_ or N_0_S_π_ conditions, in contrast to a previous report (Jiang et al., 1997a). There was also no clear pattern of same (open symbols) or opposite (filled symbols) rate-change directions for N_0_S_0_ and N_0_S_π_ conditions across frequency (i.e., thresholds were similar for upward and downward triangles). Overall, there were more neurons with the same rate-change directions than with opposite rate-change directions (more open symbols than filled symbols) between N_0_S_0_ and N_0_S_π_ conditions. Among neurons with opposite rate-change directions across conditions, more neurons had decreasing rate at threshold for the N_0_S_0_ condition (more filled downward than upward triangles). At 500 Hz, single-neuron BMLDs were close to human BMLDs at noise levels of 45 and 65 dB SPL, but not at other noise levels. At 1 kHz and above, the maximum single-neuron BMLDs were larger than human BMLDs. The maximum BMLDs were similar across noise levels, as well as across frequencies, unlike human BMLDs that decrease substantially with increasing frequency (van de Par and Kohlrausch, 1999; Goupell, 2012).

**FIGURE 9:**
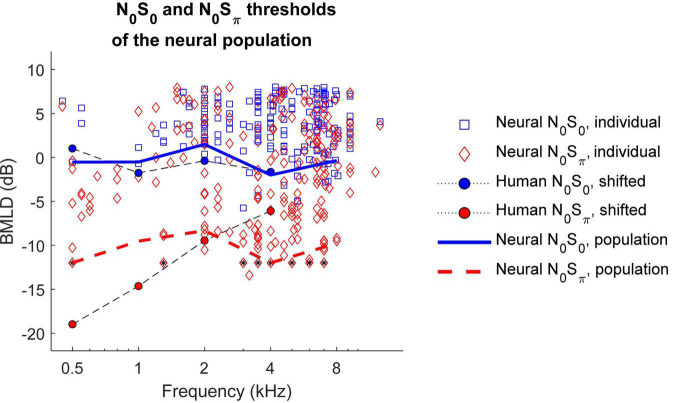
N_0_S_0_ (solid blue line) and N_0_S_π_ thresholds (dashed red line) of the neural population across frequency. Individual neural thresholds at 79.1% correct for N_0_S_0_ (blue square) and N_0_S_π_ (red diamond) conditions, for noise levels of 55–75 dB SPL are shown for all neurons with measurable thresholds above the lowest SNR tested. Symbols with a black star indicate that the threshold was lower than the lowest measured SNR. Human detection thresholds are from van de Par and Kohlrausch (1999) and shifted up by 4 dB for comparison with neural thresholds, which were computed using a higher criterion. Neural binaural masking level differences (BMLDs) had a different trend across frequency compare to human BMLDs.

To calculate BMLDs of the neural population, neural thresholds for the most sensitive subset of neurons were calculated for 0.5, 1, 2, 4, and 8 kHz for the N_0_S_0_ or N_0_S_π_ conditions. The decision to focus on the most sensitive units for this analysis, as proposed by the lower-envelope principle (Barlow et al., 1971), was based on the fact that many of the neural thresholds were significantly higher than behavioral thresholds (Figure 7). Due to the limited SNR range that was tested, many sensitive neurons were suprathreshold (greater than 70.7% correct) at the lowest tested SNR, especially for the N_0_S_π_ condition. To reduce the number of neurons for which the BMLD estimate was limited in this way, individual thresholds were recalculated using a criterion of 79.1% correct for the population-threshold results shown in Figure 9 (squares and diamonds). Individual symbols in Figure 9 represent all neurons that had thresholds above the lowest SNR tested. For each frequency, the population threshold was based on the neurons with thresholds in the lowest 10th percentile within a one-octave range centered at that frequency. Thresholds at 55–75 dB SPL had similar patterns and were plotted together in Figure 9, which shows that neural population thresholds for both N_0_S_0_ (blue solid line) and N_0_S_π_ conditions (red dashed line) did not vary across frequency. Human thresholds were moved up by 4 dB to align the means of the human and N_0_S_0_ thresholds of the population, to better compare the trend across frequency (Figure 9). Human N_0_S_π_ thresholds increase as a function of frequency, whereas thresholds of the neural population did not. Therefore, human and neural BMLDs had different trends across frequency: human BMLDs decrease with increasing frequency, whereas neural BMLDs did not. The BMLDs based on the neural population thresholds were smaller than the maximum single-neuron BMLDs, as expected due to averaging across the subsets of sensitive neurons for calculation of the population thresholds. However, the BMLDs based on either neural-population or single-neuron thresholds had similar trends across frequency.

## Discussion

In the current study, single-neuron responses to TIN stimuli were recorded in the IC for both N_0_S_0_ and N_0_S_π_ conditions over a wide range of target frequencies, as well as noise and tone levels. For the population of neurons, changes in rate due to interaural decorrelation were strongly correlated with changes in rate upon addition of an out-of-phase tone to identical noise at all noise levels.

### Comparison with previous physiological studies

There have been a limited number of physiological studies of neural responses to both N_0_S_0_ stimuli and N_0_S_π_ stimuli in the IC (Jiang et al., 1997a,b; Lane and Delgutte, 2005). The results here were most comparable to those of Jiang et al. (1997a,b), who used a tone target, as opposed to the chirp target used in Lane and Delgutte (2005). There were a few differences between the stimuli used in the current study and in Jiang et al. (1997a) that may explain differences in the results between the two studies. First, responses were only recorded for a tone frequency of 500 Hz in Jiang et al. (1997a), for neurons with a range of CFs, up to 1.5 kHz. Large differences between the tone frequency and CF would be expected to affect response properties. For example, the response of a model auditory-nerve (AN) fiber (Zilany et al., 2014) is saturated in response to a CF tone at 65 dB SPL, but not in response to a 65-dB-SPL tone one octave below CF. Therefore, when the tone frequency is far from CF, AN rates would vary with stimulus sound level, possibly a stronger cue than the relatively small change in neural fluctuations that would result from an off-CF tone. Thus, the difference between CF and target-tone frequency could explain the finding that the majority of neurons in Jiang et al. (1997a) had increasing rate with increasing SNR for the N_0_S_0_ condition, whereas many neurons in the current study had decreasing rate versus SNR.

Second, many neurons in the current study did not have measurable thresholds due to the limited range of SNRs tested, but finer steps and a wider range of SNRs were used in Jiang et al. (1997a), so thresholds were measurable for almost all neurons. However, it is worth noting that a 20-dB range of SNRs were tested in this study; thus, neurons without a measurable threshold over this SNR range were largely insensitive to addition of a tone. Thresholds for more neurons might have been measured if the SNR had been increased further, but such thresholds would likely reflect changes in response to tone levels high above behavioral thresholds, and would thus not be relevant to tone-in-noise detection.

Third, the masker in Jiang et al. (1997a) had a bandwidth from 50 Hz to 5 kHz and a level of 65 dB SPL, whereas the current study used 1/3-octave noise centered at the tone frequency, presented over a wide range of noise levels, including 65 dB SPL. The difference in masker bandwidth between studies represents a large difference in noise spectrum level: e.g., 28 dB SPL for Jiang et al. (1997a) 65 dB SPL overall level noise, versus a spectrum level of 44 dB SPL for the 500-Hz target tone tested at the overall noise level of 65 dB SPL in the current study. This difference in spectrum level would have elicited different responses in the periphery, especially at low stimulus frequencies. Even though peripheral neurons respond to a wide frequency range at high sound levels (Ruggero, 1992), the tuning is usually asymmetric and spreads more toward lower frequencies (Schmiedt, 1989). Therefore, for low-CF neurons (e.g., 1 kHz), possibly only the low frequency components of the noise masker used in Jiang et al. (1997a) effectively masked the tone. Additionally, due to non-linear cochlear compression (Robles and Ruggero, 2001), neural responses would differ for maskers having different spectral levels, though the overall level may be matched.

### The role of interaural correlation in N_0_S_π_ responses and relationship to other binaural cues

Adding an out-of-phase tone reduces the IAC (e.g., Bernstein and Trahiotis, 2017). The change in rate elicited by an out-of-phase tone was significantly correlated with the rate difference between responses to N_0_ and N_*u*_ noise (Figure 6); the large proportion of variance explained (37–69%) suggested an important role of the IAC in physiological N_0_S_π_ responses.

Results showed that both ITD-, and IAC-based cues explained a proportion of neural responses to N_0_S_π_ stimuli (maximum 34 and 69%, respectively) (Fan, 2020). The ITD-based hypothesis explained responses at low-to-medium noise levels, whereas the IAC-based hypothesis explained TIN responses at all noise levels. The IAC-based hypothesis explained a larger proportion of variance in rate responses at 65 dB SPL, at which N_0_ and N_u_ noise responses were collected. However, these cues are not independent. For example, the decreasing trend in the proportion of results explained by the ITD-based hypothesis as noise level increased could be due to the fact that envelope ITDs dominated responses of the high-CF neurons, which were the majority of the neurons in the population studied here. However, the fluctuation amplitudes in AN responses saturate (i.e., flatten) at higher sound levels, and thus binaural differences in the neural representations of the stimulus envelope would also decrease with increasing sound level, which would explain a weaker effect of envelope ITDs at high sound levels. Also, at high frequencies, IAC-cues have been proposed to be envelope-based (Durlach, 1964; Bernstein and Trahiotis, 1996).

Some effort has been made to separate the role of IAC and ITD in binaural detection (van der Heijden and Joris, 2010; Culling, 2011). Based on results from these studies, both ITD and ILD cues are proposed to contribute to interaural decorrelation. Adding an out-of-phase tone not only introduces ITDs, but also ILDs; additionally, the added binaural cues are time-varying. The dynamic range of neural responses to ILD was correlated not only to that of N_0_S_π_ responses, but also to the dynamic range of ITD responses (Figure 5). Fluctuations of ITD in an N_0_S_π_ stimulus increase with increasing tone level, whereas fluctuations of ILD first increase and then decrease as tone level increases (Zurek, 1991). Therefore, interaural decorrelation involves a nonlinear combination of ITD and ILDs cues: both ITD and ILD cues affect IAC at low tone levels, whereas at high tone levels (e.g., above 4 dB SNR), ITD cues dominate IAC. This proposed idea is consistent with a previous modeling study (Mao and Carney, 2014) in which ITD cues are shown to dominate in stimuli with low modulation depths (e.g., tone-plus-noise), and the combination of ITD and ILD cues dominate in stimuli with high modulation depths (e.g., noise). In that study, the nonlinear combination of ITD and ILD cues is described by the slope of the interaural envelope difference (SIED), whereas detection in the N_0_S_π_ condition at high frequencies has been proposed to be explained by the envelope-based IAC (Durlach, 1964; Bernstein and Trahiotis, 1996). Thus, the SIED cue is hypothesized to be a specific implementation of an envelope-based IAC in explaining N_0_S_π_ responses.

### Neural binaural masking level differences vs. human binaural masking level differences

Rate-based thresholds were estimated for both N_0_S_0_ and N_0_S_π_ conditions in order to estimate neural BMLDs over a range of frequencies and noise levels. For the N_0_S_0_ condition, the lowest rate-based thresholds across frequency could explain human detection thresholds. For the N_0_S_π_ condition, the lowest rate-based thresholds across frequency had a different trend from human detection thresholds: neural thresholds were higher (i.e., worse) than human thresholds at low frequencies, and lower (i.e., better) than human thresholds at high frequencies. Many neurons had BMLDs as large as 20 dB. BMLDs estimated based on the most sensitive units in the neural population and estimates of maximum BMLDs for single neurons only varied slightly across frequency, whereas human BMLDs decrease substantially with increasing frequency. BMLDs estimated for the neural population were shown to be slightly lower than maximum single-neuron BMLDs across all frequencies, because individual neurons with the lowest thresholds in either the N_0_S_0_ or N_0_S_π_ condition did not always have the lowest thresholds in the other condition.

Rate-based neural thresholds were similar across noise levels, consistent with human psychophysical studies (Buss et al., 2003). Human BMLDs have been shown to be minimally affected by the roving-level paradigm, in which stimulus levels randomly vary from interval to interval (Henning et al., 2005). Similar patterns of rate-based neural BMLDs across noise levels could explain the level-resistance of human listeners.

## Data availability statement

The datasets presented in this study can be found in online repositories. The names of the repository/repositories and accession number(s) can be found below: https://osf.io/kbrnw/.

## Ethics statement

The animal study was reviewed and approved by University of Rochester, University Committee on Animal Resources.

## Author contributions

LF designed and conducted the experiment, analyzed data, and wrote the manuscript. KH was involved in data analysis and provided feedback on the manuscript. LC proposed the general hypothesis, was involved in experimental design, data analysis, and edited the manuscript. All authors contributed to the article and approved the submitted version.
